# Best Management Practices for Minimizing Nitrate Leaching from Container-Grown Nurseries

**DOI:** 10.1100/tsw.2001.99

**Published:** 2001-10-17

**Authors:** Jianjun Chen, Yingfeng Huang, Russell D. Caldwell

**Affiliations:** University of Florida, IFAS, Mid-Florida Research and Education Center, Apopka 32703, USA

**Keywords:** best management practices (BMPs), containerized plants, controlled-release fertilizers, irrigation, nitrogen, nitrate leaching, potting media, water recycling, zeolite

## Abstract

Containerized plant production represents an extremely intensive agricultural practice; 40,000 to 300,000 containers may occupy one acre of surface area to which a large amount of chemical fertilizer is applied. Currently, recommended fertilizer application rates for the production of containerized nursery ornamental plants are in excess of plant requirements, and up to 50% of the applied fertilizers may run off or be leached from containers. Among the nutrients leached or allowed to runoff, nitrogen (N) is the most abundant and is of major concern as the source of ground and surface water pollution. In this report, current N fertilizer application rates for different container-grown nursery ornamental plants, the amount of nitrate leaching or runoff from containers, and the potential for nitrate contamination of ground and surface water are discussed. In contrast, our best N management practices include: (1) applying fertilizers based on plant species need; (2) improving potting medium's nutrient holding capacity using obscure mineral additives; (3) using controlled-release fertilizers; and (4) implementing zero runoff irrigation or fertigation delivery systems that significantly reduce nitrate leaching or runoff in containerized plant production and encourage dramatic changes in N management.

## INTRODUCTION

Containerized plants refer to plants grown in confined pots rather than field soil. Most plants produced in containers are those that have ornamental value such as bedding, flowering, tropical foliage, and landscape plants. Growing plants in containers does not alter the basic physiological principles involved in plant production or the genetics of the plants[1]. However, container-grown plant production differs in several aspects from other plant production systems.

A unique feature in containerized production is that plants are produced in soilless substrates called media. The vital component of media is organic matter such as pine bark, peat, coir dust, or compost. Additional materials, including perlite, vermiculite, sand, and Styrofoam, are used in various combinations with the organic materials to formulate container media. For example, Cornell Peat-lite Mix A is comprised of 50% peat and 50% vermiculite supplemented with calcium or potassium nitrate, superphosphate, ground limestone, and trace elements[2]. The media should provide water, supply nutrients, permit gas exchange to and from roots, and physically support plants. The length of time a plant can be grown or held in a container is usually limited due to the deterioration of physical and chemical properties of media and to the decrease of oxygen as the root system fills the air space of containers.

Containerized plants also must be fertilized and watered frequently on a schedule varying from weekly to daily. This is because roots are confined in a limited volume with media that retain only small amounts of water and nutrients, and these plants are often protected from natural precipitation.

Irrigation systems used for containerized plant production can be generally divided into two types: surface irrigation and subirrigation[3]. Surface irrigation includes overhead and microirrigation. Overhead irrigation sprays water over the entire bed area from nozzles that are located above the crop canopy. Microirrigation is a class of irrigation nozzles that drip, spray, or sprinkle water directly into pots[4]. Subirrigation includes ebb-and-flow and capillary mat irrigation. In ebb-and-flow systems, plants are grown on grooved ebb-and-flow trays, flooded to about an inch in depth, and allowed to absorb the solution by capillarity for a few minutes. Then, the solution is drained back to storage tanks[5]. In the capillary system, plants are placed on flat-topped benches covered with an absorbent mat. The mat is watered, and the water moves into the containers by capillary action[3]. The difference between surface irrigation and subirrigation is that the former promotes nutrient runoff between containers or leaching from containers to the soil beneath the greenhouse floor and ground, whereas the latter recycles irrigation water and ensures that zero runoff occurs.

Two types of fertilizer have been widely used in containerized plant production: water-soluble fertilizer (WSF) and controlled-release fertilizer (CRF). The WSF is applied through fertigation, i.e., fertilizer is added to irrigation water and applied via overhead irrigation, microirrigation, or subirrigation. Water-soluble fertilizers can be also incorporated into container media before transplanting. Methods of CRF applications include the incorporation of granular fertilizer into container media or the broadcast onto the surface of media substrates. It is also common practice that CRF application is supplemented with WSF.

An advantage in containerized production is that it offers opportunities to better manipulate production and postproduction processes. For example, container media can be formulated based on special needs; the amount of fertilizer application and water supply can be controlled during production; plants can be moved to different locations; marketable plants are easily handled, packaged, and transported; and finished plants are conveniently used for display purposes.

Containerized plants are high unit value crops because nurseries and greenhouse farms have the highest average net income of all agricultural commodities, nearly four times greater than traditional food and fiber farms[6]. Ornamental plants now represent the sixth largest agricultural commodity group in the U.S., with farmgate cash receipts totaling $10.94 billion in 1997. The ornamental plant industry is the fastest growing major segment of U.S. agriculture, averaging 7.1% annual growth during 1992 to 1997[7].

## NITRATE-NITROGEN LEACHING DURING CONTAINERIZED PLANT PRODUCTION

The continuous growth of the ornamental plant industry has brought about mounting concern over nutrients, especially nitrate-nitrogen ($NO_3$-N), leaching from containers and its potential contamination of surface and ground water[8,9]. Since N has a greater total influence on plant growth than most of the other essential elements[10], N is the most abundant among the elements applied to container media. Table 1 lists suggested N application rates for container-grown plants[11,12,13,14,15,16]. For example, the suggested N rates for growing Peace Lily (*Spathiphyllum*), Pothos (*Epipremnum aureum*), and Dragon Tree (*Dracaena marginata*) are 2,017, 1,755, and 2,339 kg/ha/yr, respectively, which is about 15 times higher than those recommended for many agronomic field crops. Because $NO_3$ is a major N compound in fertilizers, overfertilization combined with excessive irrigation and highly permeable media results in up to 50% of applied N leached from containers[8,9,17,18]. Ku and Hershey[9] fertigated Poinsettia (*Euphorbia pulcherrima*) using a fertilizer solution containing 210 mg/L of N and found that more than 40% and 60% of applied N was leached as leaching fraction at 0.2 and 0.4, respectively (leaching fraction is defined as the volume of leachate divided by the irrigation solution applied).

The $NO_3$-N leaching may cause ground and surface water contamination. In Europe, extremely high $NO_3$-N concentrations, up to 2,000 kg N/ha, were found in soil underlying commercial greenhouses[19]. In Connecticut, $NO_3$-N accumulation exceeding 2,300 kg/ha was recorded in soil under decades-old greenhouses[18]. A survey completed from six states, Alabama, Florida, New Jersey, North Carolina, Ohio, and Virginia, suggested that runoff $NO_3$-N levels ranged from 0.5 to 33 ppm for container nurseries using CRFs and 0.1 to 135 ppm for nurseries using CRFs supplemented with WSFs[20].

A draft for national measures to control nonpoint source pollution from agriculture was published in the Federal Register in October 2000 by the Environmental Protection Agency (EPA), which can be found at www.epa.gov/owow/nps/agmm/index.html. This EPA proposal broadens the focus of the law on the overall quality of a body of water and actually requires each state to set a limit, called "total maximum daily load," for each body of water. States would be forced to reduce "non-source" pollution such as $NO_3$-N from more diffuse sources, including agricultural and urban runoff. Consequently, containerized plant growers should be aware of the trend toward increasing stringent pollution prevention ordinances.

## SOLUTIONS TO THE NITRATE LEACHING PROBLEM

Since $NO_3$-N leaching occurs during containerized plant production, approaches to the problem should take plant species, fertilizer application rates, container media, and irrigation methods into consideration. Our proactive approaches for improving nitrogen management in the production of containerized plants, called the "Best Management Practices" (BMPs), have been developed. These include an understanding of plant species requirement for N, application of N based on plant need, the improvement of media substrates, the use of CRFs in production, and recycling of irrigation water.

### Understanding Plant Species Requirements for N

Containerized plants, like field-grown crops, should have standard growth response curves to N application rates. This curve has three well-defined regions (Fig. 1): (1) deficient range, where the growth rate increases with increasing N supply; (2) adequate range, where the growth rate reaches a maximum and remains unaffected by N supply; (3) toxic range, where the growth rate decreases with increasing N supply. To produce high quality plants while avoiding excess N application, the optimal N application rate for a plant species should be the concentration where adequate range begins (Fig. 1). Because more than 500 species and/or cultivars are currently produced in containers, the optimal N rate has not been defined for most containerized plants.

**TABLE 1**
**Suggested Nitrogen (N) Application Rates for the Production of Some of Containerized Plants in Greenhouses or Shaded Greenhouses***

| Common name | Botanical name | Nitrogen rate (kg/ha/yr) |
|---|---|---|
| Chinese Evergreen | *Aglaonema* | 1,656[11] |
| Zebra Plant | *Aphelandra squarrosa* | 1,998[11] |
| Begonia | *Begonia* x *hiemlis* | 1,425[12] |
| Peacock Plant | *Calathea makoyana* | 1,656[11] |
| Chrysanthemum | *Dendranthema grandiflora* | 1,780[13] |
| Dump Cane | *Dieffenbachia maculata* | 1,656[11] |
| Dragon Tree | *Dracaena marginata* | 2,339[11] |
| Pothos | *Epipremnum aureum* | 1,755[11] |
| Poinsettias | *Euphorbia pulcherrima* | 1,780[14] |
| Weeping Fig | *Ficus benjamina* | 2,354[11] |
| Gerbera | *Gerbera jamesonii* | 1,425[15] |
| Hibiscus | *Hibiscus rosa-sinensis* | 1,780[15] |
| New Guinea Impatiens | *Impatiens hawkeri* Bull. | 1,067[15] |
| Kalanchoe | *Kalanchoe blossfeldiana* poelln. | 1,780[15] |
| Easter Lily | *Lilium longiflorum* Thumb. | 1,425[15] |
| Prayer Plant | *Maranta leuconeura* | 1,370[11] |
| Sword Fern | *Nephrolepis exaltata* | 1,656[11] |
| Philodendron | *Philodendron selloum* | 1,998[11] |
| Azalea | *Rhododendron obtusum* | 2,237[15] |
| Peace Lily | *Spathiphyllum* | 2,017[16] |
| Arrow-Head Vine | *Syngonium podophyllum* | 1,656[11] |
| Zinnia | *Zinnia elegans* | 1,345[15] |

* Calculated based on Conover (1998)[11], Karlsson and Heins (1992) [12], Crater (1992) [13], Hartley (1992) [14], Dole and Wilkins (1999) [15], and Conover (1992) [16].

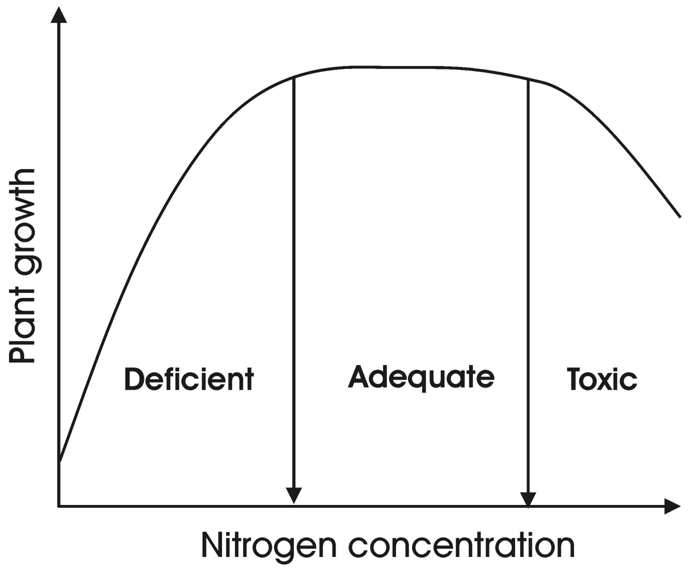

**FIGURE 1.** The relationship between N supply and plant growth. The growth (fresh weight, dry weight, or growth rate) of plants vs. increased N supply usually generates a growth response curve that has three well-defined regions: (1) deficient region, where the growth rate increases with increasing N supply; (2) adequate region, where plant growth rate reaches a maximum and remains unaffected by continuously increased N supply; (3) toxic region, where the growth rate decreases with increasing N supply.

A systematic approach for determining the optimal N application rate of tropical ornamental foliage plants has been initiated in our research program. Fig. 2 shows the growth response curve of Peace Lily and Pothos to N supply. Peace Lily had an extremely narrow adequate range for N supply, whereas Pothos exhibited a relatively wide adequate N range. Based on the curves, the optimal N application rate for Peace Lily is 2 g/15-cm pot, whereas Pothos is 2.5 g/15-cm pot annually, which are equivalent to 1,096 and 1,371 kg/ha/yr, respectively. When the suggested N rate for these two crops in Table 1 are compared to the optimal N rate derived from growth response curves, we find a 46 and 22% reduction of N supply, respectively, for Peace Lily and Pothos. This graph also illustrates that plants from different genera have different growth response curves to N supply.

The following cautions must be taken while developing the growth response curve: (1) growth response curves based on WSF may differ from those based on CRF; (2) growth curves developed from different irrigation systems may vary; and (3) different cultivars may also have different response curves. It is imperative to have growth response curves of economically important containerized plant species determined first. According to the response curves, a scientific-based optimal N application rate for the species can be recommended. It is believed that using this scientific-based optimal N rate in containerized plant production could reduce N application by at least 20% while maintaining plant quality.

## Improving Container Media

Since the commercialization of container media after World War II, media components have been predominantly pine bark, peat, vermiculite, and perlite. Components newly introduced are coconut coir and polymer gel. Little attempt has been made so far to improving nutrient holding capacity of container media. Our recent studies on chemical property changes in container media indicated that with continuous fertigation the cation exchange capacity, a parameter for measuring nutrient holding capacity, and pH of media significantly decreased. To improve media nutrient holding capacity, we evaluated various soil amendments and identified that zeolite incorporation can improve nutrient holding capacities of media. Zeolites are aluminosilicate minerals, possessing channels and interconnecting cavities in their tetrahedral frameworks. Crystalline zeolites are the most effective ion exchangers, with capacities ranging from 1.5 to 5.5 meq/g (anhydrous) and are able to adsorb up to 30% of their dry weight. More than 50 naturally occurring zeolites have been identified, and 100 have been synthesized[21]. Many of them are available commercially.

In a recent study, six zeolites were individually mixed with a peat and bark formulated media at 5, 10, and 20 g/10-cm pot to grow Pink Splash (*Hypoestes phyllostachya*). A solution containing a WSF with N at a concentration of 200 mg/L was applied at 100 mL per pot weekly. Results demonstrated that zeolite-amended media leached 30 to 90% less ammonium depending on zeolite species and up to 50% less nitrate than the control media three months after transplanting (Table 2)[22]. Zeolite amended media had no adverse effects on plant growth, but rather promoted plant growth compared to control media[23].

Research on amended zeolites interacting with applied CRFs is underway. Urea is a major source of N in CRFs. After application, urea is rapidly transformed, through the activity of the enzyme unease, to ammonia, which is converted to nitrite and finally nitrates. The presence of zeolites in media will keep nitrogen in the ammonia form, thereby increasing the efficiency of the nitrogen applied and reducing leaching.

## Using Controlled-Release Fertilizers

The most widely used CRFs in containerized plant production are granules or prills of fertilizers coated with a semi-permeable

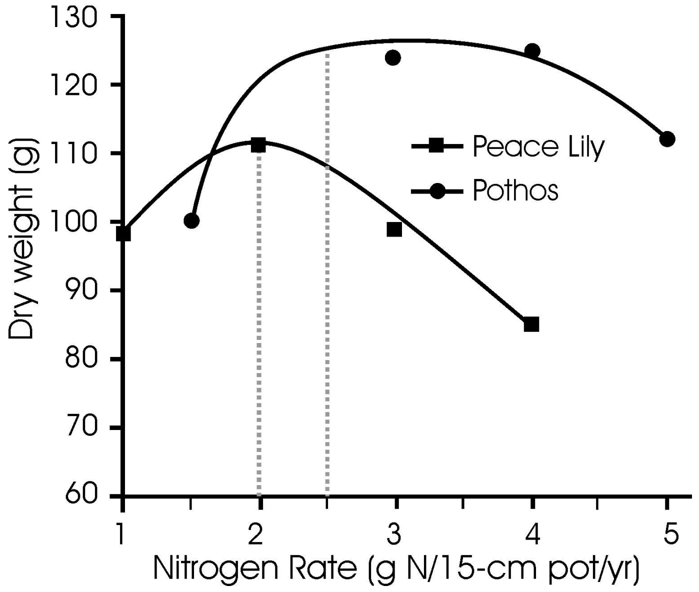

**FIGURE 2.** The growth response of Peace Lily (*Spathiphyllum*) and Pothos (*Epipremnum aureum*) to N supply. Peace Lily exhibited a narrow adequate range for N, whereas Pothos showed a relatively wide adequate N range. Based on the growth response curves, the optimal N rate for Peace Lily and Pothos should respectively be 2.0 and 2.5 g N per 15-cm pot annually as indicated by dotted lines.

**TABLE 2**
**Average Ammonium–Nitrogen ($NH_4$-N) and Nitrate–Nitrogen ($NO_3$-N) Concentrations in Leachates of Control and Zeolite-Incorporated Soilless Media 9 Weeks after Transplanting and Average Dry Weights of Pink Splash (*Hypoestes phyllostachya*) Produced in the Media***

| Media | $NH_4$-N (mg/kg) | $NO_3$-N (mg/kg) | Dry weight (g) |
|---|---|---|---|
| Control | 132.6 | 228.4 | 2.1 |
| A | 7.7 | 397.7 | 2.5 |
| CH | 92.5 | 192.7 | 2.7 |
| IE | 53.7 | 251.5 | 2.8 |
| SC | 115.2 | 213.2 | 3.4 |
| X | 41.4 | 307.5 | 2.8 |
| XY | 82.6 | 109.3 | 3.3 |

* Six zeolites (A, CH, IE, SC, X, and XY) were mixed respectively with the control medium (50% peat + 50% pine bark) at 20 g of zeolite per pot (10-cm in diameter). Seedlings of Pink Splash were transplanted into pots with 8 plants per pot. Peter's water-soluble fertilizer (24.0N-4.4P-16.6K) solution containing 200 mg/L N was fertigated weekly at 100 mL per pot. Leachates were collected weekly. Data presented in this table were the final samples. Plant shoots were harvested nine weeks after transplanting, and dry weights were determined.

layer. After application, water penetrates the coating and dissolves the nutrients inside, gradually releasing the elements into container media. CRFs presumably release their nutrients steadily day by day, instead of in peaks, thus, a constant flow of nutrients becomes available to plant roots. Plants fertilized with CRFs typically have less $NO_3$-N leaching than plants fertilized with WSF. Broschat[8] compared total $NO_3$-N leached per container from liquid fertilizer with CRFs over six months and found that media fertilized with CRF leached 28 to 40% less $NO_3$-N than those fertilized with WSF. Currently, a majority of Florida's nurseries use CRFs in container-growth plant production[24].

However, other studies showed that CRFs resulted in $NO_3$-N leaching the same as delivering fertilizer in irrigation water[25]. The variable performance of CRFs could be due to the differences in fertilizer coating technology, different methods of application, or different leaching fractions. Recent studies[8,9,26,27] suggested that the use of CRFs significantly reduced $NO_3$-N leaching in containerized plant production if leaching fractions were at 0.2 or less. In addition, the development of new technologies for better controlling nutrient release will lead to reduce $NO_3$-N leaching.

## Irrigation System Improvement

As water and fertilizer are interrelated in containerized plant production, one promising way to avoid N runoff or leaching into groundwater is to use zero runoff subirrigation. Growers in Florida adopting either ebb-and-flow or capillary mat irrigation reported 20% reduction of fertilizer use and 75% reduction of water consumption in containerized plant production. The disadvantages of subirrigation include a greater initial capital outlay, it is limited to growing one crop and container size per irrigation zone, and salinity problems may occur if not managed properly.

Another irrigation system, which can achieve minimal runoff and less salt buildup in media, is to use surface irrigation systems, but capture, retain, and recycle the runoff and stormwater within the boundaries of the production facility[19]. This is exemplified by whole greenhouse/nursery recycling systems, called the total nursery recycling system[28]. This recycling system includes: (1) stormwater and/or irrigation runoff collection; (2) sedimentation, flocculation, filtration, and disinfection, if necessary; and (3) irrigation. Skimina[29] tested more than 100 species of landscape ornamental plants using this system and found that the range of plant growth response was 73 to 171% relative to control plants.

However, limited nurseries have used this total nursery recycle system for the production of greenhouse containerized plants. Growers were concerned about the feasibility and reliability of the water sources for the production of high quality plants. To address this concern, we evaluated a total of 30 container-grown plant species (22 foliage plants and 8 bedding plants) using water collected from rain and/or irrigation runoff, well water under ebb-and-flow, and overhead irrigation systems. All plants at the time of harvest were of marketable sizes and salable quality independent of water sources. No disease incidences or growth disorders related to water sources were observed. Results indicate that water collected from rain and/or irrigation runoff can be alternative sources of water for irrigation of greenhouse containerized plants[30]. Table 3 shows the average amount of fertilizers and water used per pot for the production of 1,080 containerized plants. Ebb-and-flow used 3.5 times less of water

**TABLE 3**
**Average Amounts of Total Water and Fertilizers Used per Pot for the Production of 1,080 Pots of Bedding and Foliage Plants***

| Irrigation Method | Water (L/pot) | CRF (g/pot) | WSF (g/pot) | $NO_3$-N Leaching (mg/pot) |
|---|---|---|---|---|
| Overhead | 35.5 | 5.0 | | 450.5 |
| Ebb-and-flow | 10.2 | | 3.7 | 0.0 |

* Thirty plant species and/or cultivars (22 foliage plants and 8 bedding plants) were grown in a soilless medium irrigated through ebb-and-flow and overhead system using water collected from rain and/or irrigation runoff and well water. Peter's water-soluble fertilizer 24.0N-3.5P-13.3K (24-8-16) was used for fertigating plants grown on ebb-and-flow trays, and Scotts' controlled-release fertilizer 18.0N-2.6P-10.0K (Osmocote 18-6-12) was applied onto the surface of pots irrigated overhead. A total of 1,080 pots of plants were produced over two and half years of evaluation. Data presented here are mean total amounts of water regardless of sources and fertilizers per pots used for the production of 1,080 plants from transplanting to the market.

than overhead irrigation and had no nutrient leaching from containers. Overhead irrigation with a CRF had a mean total of 450.5 mg of $NO_3$-N leaching from each container, but there was no N runoff to the soil beneath the greenhouse floor and ground since water was captured in the implemented total recycling system. The total nursery recycle system has advantages of: (1) being simple and affordable; (2) having zero runoff, thus, no nutrient coursing, particularly nitrate and phosphorus, into aquifers; (3) water conservation; (4) nutrient recycling; and (5) the production of high quality plants.

## CONCLUSION

Containerized plants are grown in confined media that are highly permeable and have low water and nutrient holding capacities. Due to a large number of plant species being produced in containers, the optimal N application rates for most plants have not been developed. Instead, a large amount of N is applied, which is far more than plants need. As a result, up to 50% of applied N may run off between or leach from containers to the soil beneath the greenhouse floor and ground or into groundwater. Ingestion of nitrate from drinking water can cause various health problems[31,32].

Solutions to prevent or reduce $NO_3$-N runoff or leaching are: (1) applying N based on plant species-specific needs; (2) improving container media; (3) using CFRs in production; and (4) implementing the total nursery recycle system. Current research has shown that improving each of these areas can significantly reduce $NO_3$-N leaching or runoff. Future studies will define the best practices in each area and integrate them into the BMP program for minimizing $NO_3$-N leaching during containerized plant production.

## ACKNOWLEDGMENTS

Florida Agricultural Experiment Station Journal Series No. R-08327. This work was supported in part by the St. Johns River Water Management District, FL, the University of Florida, IFAS Extension Water Quality, and USDA Smith-Lever Water Quality Extension Awards.

| | | |
|---|---|---|
| Received: | July | 7, 2001 |
| Revised: | September | 11, 2001 |
| Accepted: | September | 12, 2001 |
| Published: | October | 17, 2001 |
